# Anamnestic risk factor evaluation of patients carrying carbapenem-resistant Enterobacterales and/or Acinetobacter baumannii – impact on infection control management at a German University Hospital 

**DOI:** 10.3205/dgkh000340

**Published:** 2020-04-23

**Authors:** Franziska Hofmann, Ursel Heudorf, Katrin Steul, Thomas A Wichelhaus, Silke Besier, Michael Hogardt, Daniel Hack, Elvira Steinmann, Volkhard A J Kempf, Claudia Reinheimer

**Affiliations:** 1 Institute of Medical Microbiology and Infection Control, University Hospital Frankfurt, Germany; 2 University Center for Infectious Diseases, University Hospital Frankfurt, Germany; 3 University Center of Competence for Infection Control, State of Hesse, Germany; 4 Public Health Department of the City of Frankfurt/Main, Germany

**Keywords:** carbapenem resistance, Enterobacterales, A. baumannii, risk factors, infection control management

## Abstract

**Background:** Carbapenem-resistant *Enterobacterales* and *Acinetobacter* *baumannii* are of major concern in terms of infection prevention and control. This study evaluated factors that may increase the frequency of *Enterobacterales* and *A. baumannii* with carbapenem resistance (CR) in patients admitted to a German University Hospital for implementation of optimized infection control management.

**Methods:** A five-year-retrospective epidemiological cohort analysis was conducted on anamnestic risk factors for carrying *Enterobacterales* and/or *A. baumannii* with CR in patients who were first tested positive for these species at University Hospital Frankfurt (UHF) between January 2013 and June 2018.

**Results:** 364 patients were tested positive for *Enterobacterales* and/or *A. baumannii* with CR, resulting in n=400 bacterial isolates in total, with *Klebsiella pneumoniae* being the most frequently detected species (n=146/400; 36.5%; 95% confidence interval: 31.8–41.4). In patients who were tested positive for *Enterobacterales* and/or *A. baumannii* with CR, any hospital stay within the previous 12 months was the most frequently reported common factor (n=275/364; 75.5%; 70.8–79.9).

**Conclusion:** A hospital stay within the previous 12 months, including hospitals in Germany and abroad, is a frequent characteristic of patients who tested positive for *Enterobacterales* and/or *A. baumannii* with CR. Upon admission, any previous hospital stay of the given patient within the previous 12 months should be determined. Infection control strategies such as screening measures need to be adapted to these patient groups in hospital settings. In order to reflect the varying determinants in “nosocomial” cases in greater detail, the existing criteria used to characterize “nosocomial detection” of gram-negative bacteria with CR should be reviewed.

## Zusammenfassung

**Hintergrund:** *Enterobacterales* und/oder *Acinetobacter* *baumannii* mit Carbapenem-Resistenz (CR) stellen in Kliniken eine enorme medizinische Herausforderung dar. Die vorliegende Studie untersucht anamnestische Charakteristika von Patienten des Universitätsklinikums Frankfurt am Main (UKF), die zum Risiko einer Trägerschaft von *Enterobacterales* und/oder *A. baumannii* mit CR beitragen. Ziel der Untersuchung ist die Etablierung einer zielgerichteten Strategie zur Infektionskontrolle.

**Methode:** Retrospektive epidemiologischen Kohortenanalyse bei Patienten, die zwischen Januar 2013 und Juni 2018 am UKF positiv auf mindestens eine Spezies *Enterobacterales* und/oder *A. baumannii* mit CR getestet wurden.

**Ergebnisse:** Innerhalb des Untersuchungszeitraums wurden bei 364 Patienten 400 Isolate von *Enterobacterales* und/oder *A. baumannii* mit CR nachgewiesen. Hiervon am häufigsten vertreten war *Klebsiella pneumoniae* (n=146/400; 36,5%; 95%-Konfidenzintervall: 31,8–41,4). Die anamnestische Angabe mindestens eines Krankenhausaufenthalts „innerhalb der letzten 12 Monate“ wurde am häufigsten berichtet (n=275/364; 75,5%; 70,8–79,9).

**Zusammenfassung:** Da sich ein Krankenhausaufenthalt innerhalb der letzten 12 Monate mit Nachweis von *Enterobacterales* und/oder *A. baumannii* mit CR als das am häufigsten berichtete Risikomerkmal herausstellte, sollte das bei Aufnahme eines Patienten sorgfältig abgefragt werden. Krankenhaushygienische Strategien zur Infektionsprävention, z.B. Implementierung von Screeningprotokollen für Patienten mit diesem Risikofaktor, können hilfreich sein und sollten situationsadaptiert eingesetzt werden. Um die derzeit als „nosokomiale Nachweise“ zu bewertenden Fälle richtig darstellen zu können, ist eine Überarbeitung der derzeitigen Definitionskriterien notwendig.

## Introduction

The dramatic worldwide spread of multidrug-resistant organisms (MDRO), and that of multidrug-resistant gram-negative organisms with carbapenem-resistance (MDRGN with CR) in particular, is an issue of major concern in terms of epidemiology and infection control [1], [2], [3], [4], [5], [6], [7]. Several factors contributing to MDRGN’s global spread have been determined, such as international travel [8], [9], [10], refugee history [11], [12], [13], [14], medical tourism or medical pre-treatment abroad [11], [14], [15]. Despite the knowledge of such factors and the implementation of strict infection control measures, such as isolation strategies [11], [12], MDRGN remain a major health challenge in hospitals. This suggests that several other factors might additionally contribute to the massive spread of MDRGN with CR. On average, University Hospital Frankfurt, Germany (UHF) deals with around 75 cases of newly detected *Enterobacterales* and *A. baumannii* with resistance to carbapenems (CR) every year. This high number of MDRGN with CR might particularly be attributed to the UHF’s direct vicinity to Frankfurt International Airport, whence a relevant number of patients reporting a stay abroad are admitted. 

In order to maintain a firm infection control strategy, we evaluated factors that might additionally pose a risk factor of carrying *Enterobacterales* and/or *A. baumannii* with CR detected in patients admitted to UHF over a five-year period. Data were regularly obtained in accordance with mandatory reporting stipulated by specific regulations of the federal state of Hesse and Germany [16], [17], [18], [19], [20]. They form the basis of discussion within a local MDRO network (Rhine-Main-Network, Hesse [21]) to build trans-hospital knowledge in the regional infection control departments. This study evaluated patients who were tested positive for *Enterobacterales* or *A. baumannii* with CR at UHF for anamnestic risk factors, e.g*.*, being a resident of a nursing home or having a history of hospital stays in Germany or abroad, as well as previous non-medical (tourism) stays abroad.

## Materials and methods

### Definition of multidrug-resistant gram-negative bacteria with resistance to carbapenems (MDRGN with CR) 

MDRGN are defined as *“Enterobacterales* with extended spectrum beta-lactamase (ESBL)–phenotype as well as *Enterobacterales*, and *Acinetobacter baumannii* resistant against piperacillin, any 3^rd^/4^th^ generation cephalosporin, and fluoroquinolones ± carbapenems”, as previously described [11], [12]. In case of carbapenem-resistance, the addendum “CR” is given. 

### Infection control surveillance and German infection protection law

In Germany, the infection protection law (Infektionsschutzgesetz; IfSG) determines various aspects of infection control and epidemiological surveillance, and mandatory reporting of certain infections or infectious agents [20]. Additionally, the Federal States of Germany may require further mandatory reporting. Hence, in 2011, the federal state of Hesse made reporting of multidrug-resistant gram-negative species *(Enterobacterales*, *A. baumannii* and *Pseudomonas* spp.) with CR mandatory, regardless of the resistance mechanism or the nature of the patient sample from which the respective species was obtained [16]. In 2013, *Pseudomonas aeruginosa* was omitted from this regulation [17]. Based on the Hessian experience, the obligation to report *Enterobacterales* and *A. baumannii* with CR was introduced across Germany [18], [19], [20]. In Hesse, mandatory MDRGN notification is given by using a standardized questionnaire [18]. Data obtained by the questionnaire encompass information on the patient’s current residency status (e.g., in a nursing home), sojourns outside Germany or hospital stays within the preceding 12 months, i.a. grouping into colonization versus infection and in hospital-acquired versus community-acquired (definitions given below) is also required as part of the notification to the responsible public health authority. Thus, Hesse was the first German federal state to introduce the obligation to report carbapenem-resistant organisms, resulting in long-standing experience in reporting and management of these pathogens in Hesse, especially in UHF. 

### Screening procedure at UHF

German hospitals need to adhere to an infection control strategy which describes actions necessary to prevent the transmission of harmful organisms during the patients’ hospital stay. This legal obligation is based on the German infection protection law; risk-adapted screening is recommended by KRINKO [7], [20], as mentioned above, and is therefore mandatory for the hospital’s employees. At UHF, this demand is met and documented in the UHF’s infection control strategy, as previously described [11]. Upon admission, screening for MDRGN *(Enterobacterales* and *A. baumannii*) includes rectal and throat samples as well as swabs from wounds, if the patient has wounds, as well as tracheal secretion, if patient is intubated.

### Colonization versus infection and case definition

Colonization (CO) and infection (INF) were recorded in the questionnaire [18]. CO was entered if *Enterobacterales* and/or* A. baumannii* with CR were detected solely in screening samples, e.g., rectal, throat or cutaneous swabs, and the patient was free of any local or systemic infection signs. Following the definition by the hospital infection surveillance system (Krankenhaus-Infektions-Surveillance-System, KISS) of the National Reference Center for Surveillance of Nosocomial Infections, Berlin, Germany (*Nationales Referenzzentrum für Surveillance von nosokomialen Infektionen*), INF was entered if *Enterobacterales* or *A. baumannii* with CR were found in primarily sterile materials (e.g., blood), pus, wounds, and certain infection symptoms (e.g, fever) or associated with typical laboratory results (e.g., leukocytosis, microbiological detection of an infectious agent) as well as corresponding results from imaging procedures (e.g., X-ray, computed tomography, nuclear magnetic resonance tomography, ultrasound) and endoscopic examinations [22]. Further, also following the KISS definition, CO or INF are labelled as “community-acquired” (CA), if the pathogen is detected within the first three days after admittance, with the day of admittance being day one [22]. If the pathogen is detected later than three days after admittance, with the day of admittance being the first day, CO/INF are labelled as “nosocomial” which is commonly meant to be “hospital-acquired” (HA). 

### Patients and samples

In this retrospective study, n=364 patients were analyzed. These patients were tested positive for the first time for *Enterobacterales* and/or* A. baumannii* with CR upon being admitted to UHF between January 1^st^, 2013 and June 15^th^, 2018. Patients’ data were obtained from the notification questionnaire [18], as indicated above, as well as from the patients’ digital records.

### Detection of MDRGN and molecular resistance analysis

Laboratory testing was performed under strict quality-controlled criteria (laboratory accreditation according to ISO 15189:2007 standards) at the Institute for Medical Microbiology and Infection Control, University Hospital Frankfurt, Germany. Samples were collected using culture swabs as well as Amies collection and transport medium (Hain Lifescience, Nehren, Germany) and streaked onto selective CHROMagarTM ESBL plates (Mast Diagnostica, Paris, France). Identification of presumed MDRGN species and antibiotic susceptibility testing were performed as previously described [11]. Carbapenemase encoding genes were detected via PCR analysis and subsequent sequencing from carbapenem-resistant *Enterobacterales*, including the *bla* genes for carbapenemases NDM, VIM, IMP, OXA–48–like and KPC as well as OXA–23, OXA–24 (including subtypes), and OXA–58 and NDM as well as species-specific OXA-51 for *A. baumannii* [11], [23], [24]. 

### Statistical analysis

The biostatistical data file from the University of Münster, Germany, was used for statistical analyses of the pseudonymized data [25]. 95% confidence intervals (95%CI) were calculated based on binomial distribution; p-values (2-tailed) of p≤0.05 were considered statistically significant. 

## Results

### General characteristics of the study cohort

Between January 1^st^, 2013 and June 15^th^, 2018, a total of 364 patients were tested positive for the first time for *Enterobacterales* and *A. baumannii* with CR at UHF. N=238/364 were male (65.4%). The mean age was 58.5 years (standard deviation 21.3), with a median of 65 years. 

### Notifications within the observation period 

**Figure 1:**
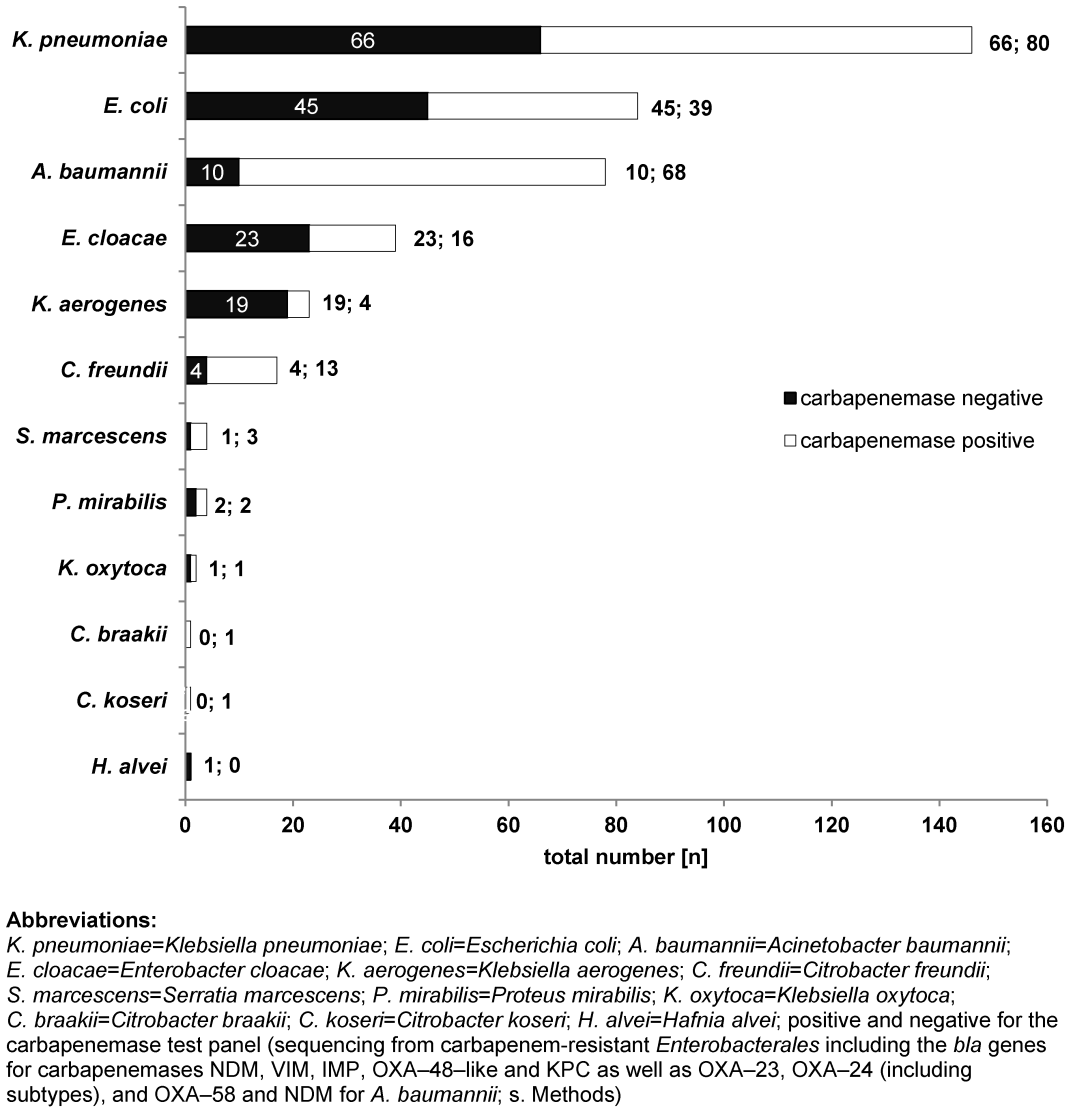
Number of *Enterobacterales* and *A. baumannii* with CR detected in UHF reported to the Public Health Authority Frankfurt am Main, Germany (n=364; as of January 1^st^, 2013 and June 15^th^, 2018)

**Figure 2:**
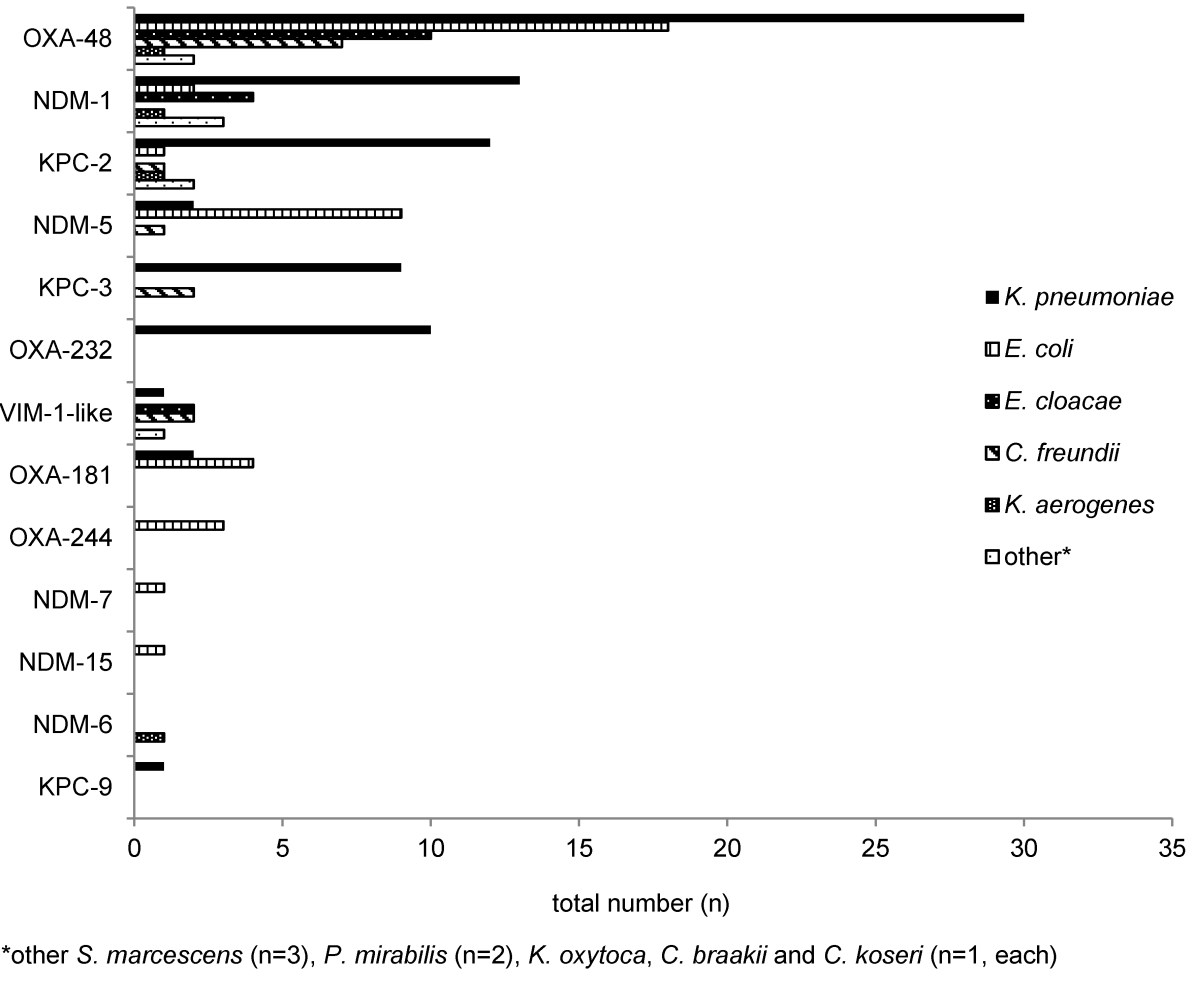
Carbapenemases detected in *Enterobacterales* with CR (n=160) during the observation period

**Figure 3:**
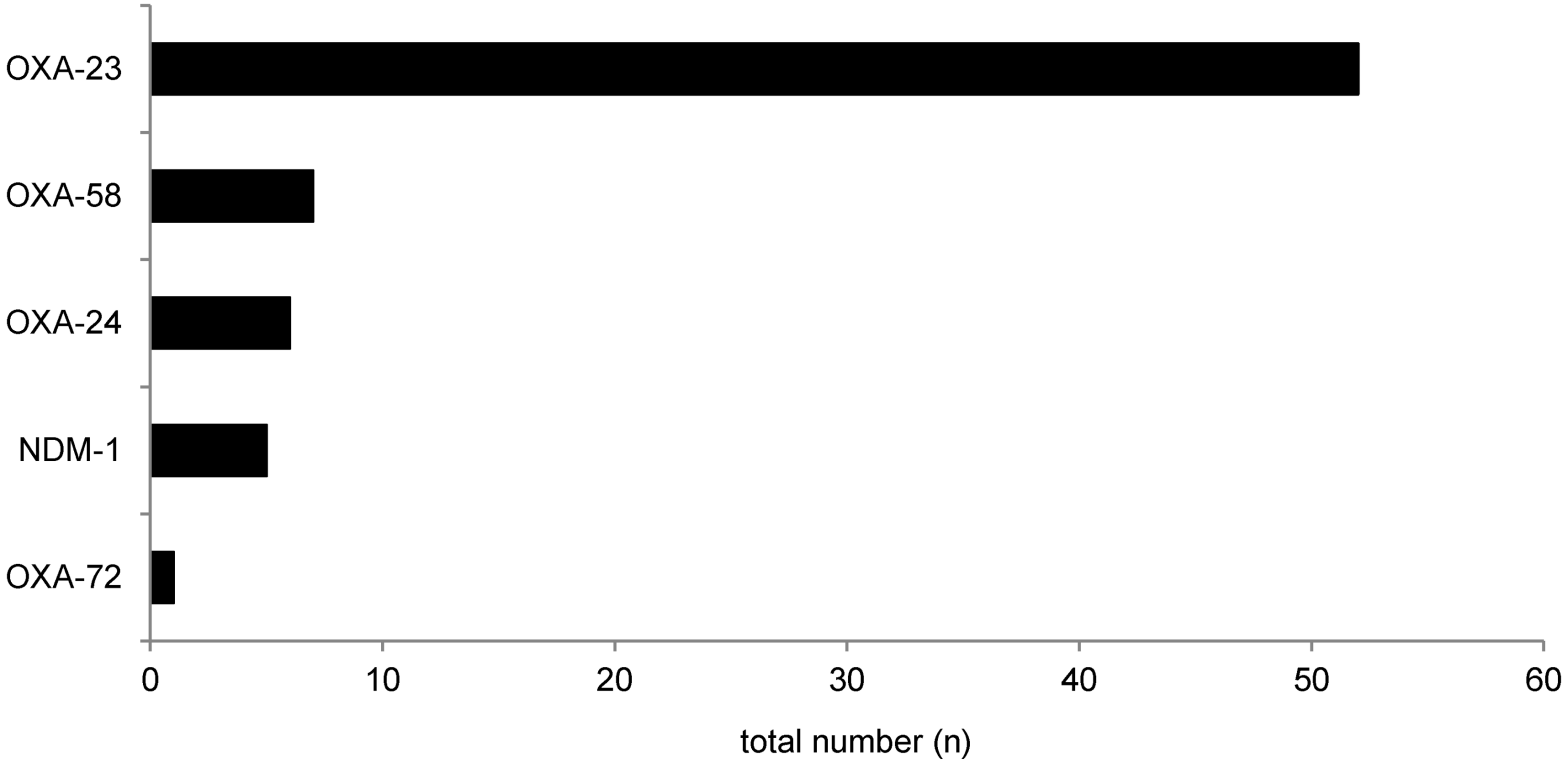
Carbapenemases detected in *A. baumannii* with CR (n=71)

N=28/364 patients were tested positive for more than one species of *Enterobacterales* and/or *A. baumannii* with CR, resulting in a total number of n=400 isolates reported to the public health authority in Frankfurt am Main, Germany. Of these, *K. pneumoniae* with CR was the most frequently detected species, n=146/400 (36.5%), followed by *E. coli* with CR, n=84/400 (21.0%), and *A. baumannii* n=78/400 (19.5%) (Figure 1 (Fig. 1)). The types of carbapenemases identified in the study population are given in Figure 2 (Fig. 2) and Figure 3 (Fig. 3). One (n=1) *A. baumannii* isolate was found positive for three carbapenemases (NDM-1 + OXA-23 + OXA-58) and one (n=1)* A. baumannii* isolate was tested positive for two carbapenemases (OXA-23 + OXA-58). 

### Case characteristics 

**Figure 4:**
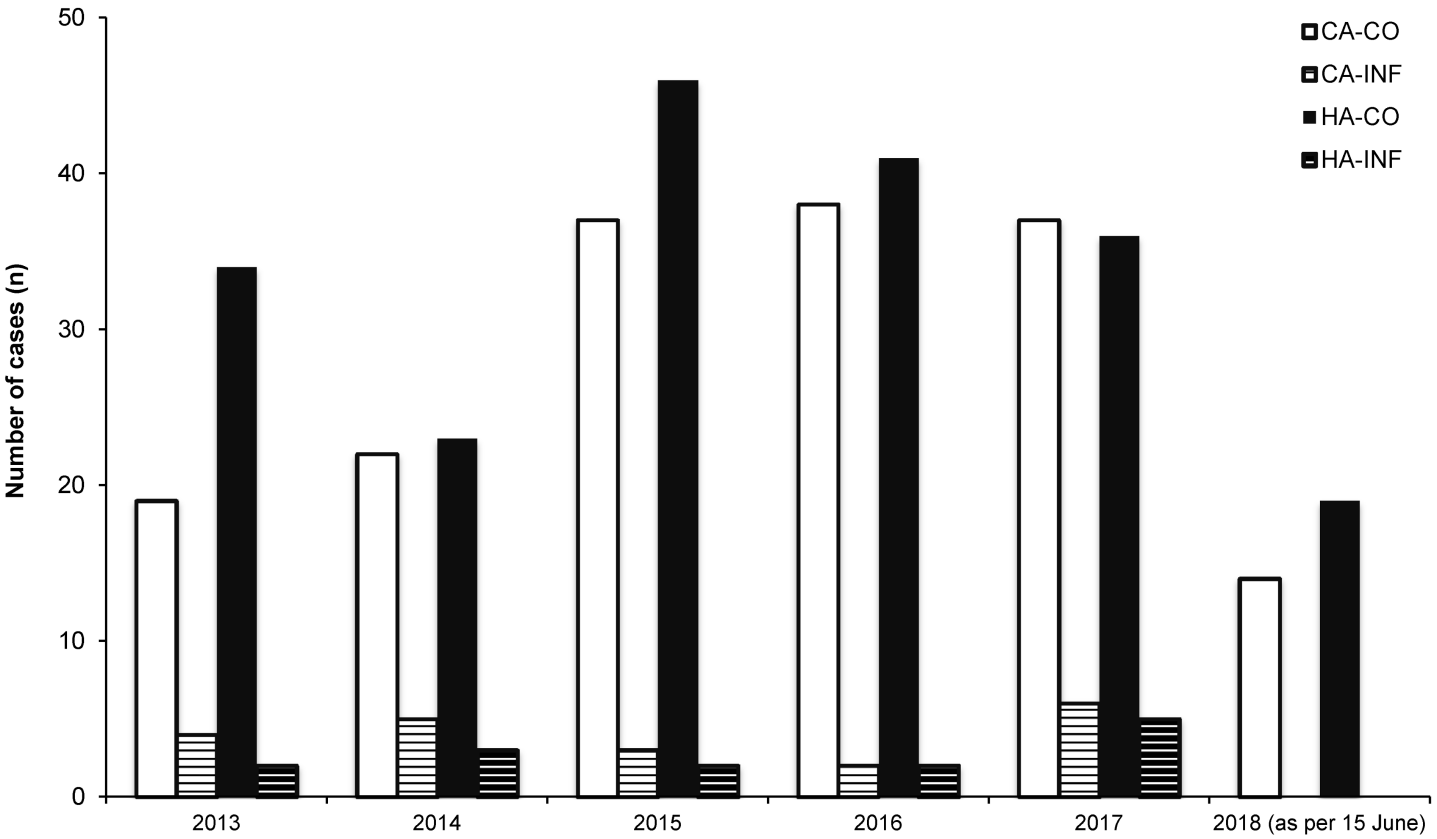
Number of “community acquired and colonized” (HA-CO), CA and infected (CA-INF), hospital-acquired and colonized (HA-CO) as well as HA and infected (HA-INF) cases between January 2013 and June 2018

In total, n=187/400 (46.8%) isolates were found to be community-acquired (CA) and n=213/400 (53.3%) were hospital-acquired (HA), based on the definition by KISS ([22]; see methods). Regarding CA isolates, n=167/187 (89.3%) and n=20/187 (10.7%) isolates were obtained from screening material (“colonization”; CA-CO) and from invasive materials (“infection”; CA-INF), respectively. In HA, n=199/213 (93.4%) of the patients were colonized (HA-CO), whereas n=14/213 (6.6%) were infected (HA-INF). Further details are shown in Figure 4 (Fig. 4).

### Enterobacterales and A. baumannii with CR: anamnestic evaluation 

The anamnestic factors found in the history of patients who were tested positive for *Enterobacterales* and/or *A. baumannii* with CR are summarized in Table 1 (Tab. 1). A selection of multiple items was possible. In terms of residential status, patients who were tested positive for *Enterobacterales* and/or *A. baumannii* with CR most frequently reported “own household” with n=338/364 (92.9%; 89.7–95.3). n=275 patients reported n=291 stays in hospital within the previous 12 months. Of these, n=99 stays were at hospitals abroad and n=192 stays in a German hospital within the previous 12 months. Due to multiple answers, both admittance to a German hospital and a hospital outside of Germany within the previous 12 months was reported by n=16 patients. “Stay in foreign country without any contact to local health care system (HCS) within the previous 12 months” was reported by n=28/364 (7.7%; 5.2–10.9) patients.

## Discussion

Multidrug-resistant gram-negative bacteria remain a critical issue in terms of infection control. Deeper knowledge of their epidemiology, associated risk factors, and effective infection control strategies is needed in order to prevent the spread of these challenging pathogens. The anamnestic background of patients who were tested positive for *Enterobacterales* and/or *A. baumannii* with CR could give valuable insight into the epidemiology of these species and identify markers, in order to develop optimized, risk-adapted infection control management.

In more than a half of the isolates in the present study, at least one carbapenemase (n=228/400; 57.0%) was identified. Of these, n=160/322 (49.7%) and n=68/78 (87.2%) *Enterobacterales* and/or *A. baumannii*, respectively, were found to be positive for a carbapenemase of the tested panel. *K. pneumoniae* with CR was the most frequently detected species, followed by *E. coli* with CR and *A. baumannii* with CR (Figure 1 (Fig. 1)). As demonstrated in Figure 2 (Fig. 2) and Figure 3 (Fig. 3), n=13 different carbapenemases were detected in *Enterobacterales* and five different carbapenemases in *A. baumannii* isolates, partly in combination with other carbapenemases. Of the carbapenemases identified, OXA-48-like and OXA-23 were most frequently found in *Enterobacterales* and *A. baumannii*, respectively. This is congruent with data obtained from the Rhine-Main-area and the German National Reference Laboratory (NRC) for multidrug-resistant gram-negative bacteria [26], [27]. The possibility must be mentioned that isolates which were tested negative for carbapenemase may nevertheless be positive for other carbapenemases which were not included in our laboratory panel. In addition, altered expression of porins or function of efflux pumps can also result in carbapenemase-negative carbapenem resistance [28].

Interestingly, VIM-1, which is the second most frequently detected carbapenemase in *Enterobacteriaceae* in Germany [27], ranked rather low in our study (n=6 isolates). Concerning KPC-2 and KPC-3 as well as NDM-1 and NDM-5, however, data from the NRC [26] and our study (Figure 2 (Fig. 2) and Figure 3 (Fig. 3)) largely match. The large proportion of MDRGN tested positive for carbapenemases and the occurrence of isolates that were tested positive for more than one carbapenemase particularly emphasizes the hazard potential of these pathogens. Clearly, the appearance of MDRGN expressing several different carbapenemases calls for infection control strategies to prevent the development of a new hospital health threat.

Within the study’s observation period, n=400 isolates of MDRGN with CR were reported, which is an estimated number of ca. n=75 cases annually at UHF. In comparison, data on *Enterobacterales* and *A. baumannii* with CR reported by all Frankfurt hospitals to the municipal public health department of Frankfurt am Main, Hesse, Germany show a total number of n=559 notifications for *Enterobacterales* and *A. baumannii* with CR between April 2012 and December 2015 [27], which is an estimated 150 cases annually. This highlights the glaringly high number of *Enterobacterales* and *A. baumannii* with CR UHF has to deal with. With regard to the patient population at university hospitals, this high number of patients carrying MDRGN with CR is not surprising. For example, distinct and harmonized screening procedures for MDRO, critically ill patients suffering from complex diseases with a long history of pre-treatment in other hospitals before being transferred to our university hospital, and patients admitted after pre-treatment abroad may greatly contribute to a high prevalence of MDRGN with CR at any university hospital. In turn, the number of HA-INF within the observation period was low, n=14/214 (Figure 4 (Fig. 4)), indicating effective infection control management.

Regarding the patients’ residential status, the anamnestic status “own household” should be interpreted carefully. Although people living in their own household may generally be healthier compared to people living in a nursing home, who may have a higher risk of carrying health-care-associated MDRGN with CR, the former groups are suggested to have travelled more, for instance. This in turn might be associated with travel-associated factors, e.g., a hospital stay abroad, to be at higher risk of testing positive for MDRGN with CR. Regarding our data, 92.9% (89.7–95.3) of the patients who were tested positive for MDRGN with CR reported living in their “own household” compared to a significantly lower percentage, 4.7% (2.7–7.4), reporting “nursing home” as their residential status. However, the number of patients who were tested negative for MDRGN with CR and reported living in their own household versus a nursing home has not been evaluated. Thus, because these data were not available in our study, a reliable conclusion about this aspect cannot be given.

The study patients who were tested positive for Enterobacterales and/or *A. baumannii* with CR reported n=275 stays in a hospital within the previous 12 months, indicating that a previous hospitalization is strongly associated with carrying *Enterobacterales* and/or *A. baumannii with* CR, which has also been suggested previously [11], [27], [29], [30], [31]. Of this patient group, the majority of hospital stays (n=192/275; 69.8%; 64.0–75.2) were in a German hospital within the previous 12 months, indicating a key characteristic among patients carrying *Enterobacterales* and/or *A. baumannii* with CR. To support this conclusion, however, data on the basic population of all patients being admitted to UHF reporting a stay in a German hospital within the previous 12 months would be necessary, especially in order to compare this to any hospital stay abroad (n=99/275; 36.0%; 30.3–42.0). The average number of patients who are directly transferred from any German hospital to UHF amounts to at least 2,000 *per anno*. This number, however, might only be a fraction of the total patient number reporting a stay in a German hospital within the previous 12 months; an epidemiological conclusion can only be drawn to a limited extent. 

Interestingly, 7.7% (5.2–10.9) of the patients who were tested positive for *Enterobacterales* and/or *A. baumannii* with CR reported a “stay in a foreign country without any contact to local HCS”, which itself has also been identified as a risk factor for carrying MDRGN, [9], [10], [11], [14], [15], [32], [33], [34], [35], [36], [37]. In order to investigate this patient group, however, data on the countries they visited as well as their duration of stay in the respective countries would be needed. In an ongoing project (different patient cohort, observation period January 2013–January 2017), n=23 patients were identified reporting a stay abroad in the UN region Southern Asia without any contact to local health care systems. Of these, India (n=15) and Pakistan (n=3) were the most frequently reported countries visited. Of these, n=17/23 patients (73.9%; 51.6–89.8) were tested positive for MDRGN. Three patients were found positive for n=3 MDRGN + CR isolates in total (with n=1 isolate in each patient), with all of them reporting a stay in India. The types of carbapenemases detected in these isolates were NDM-5 and OXA-181 (n=1 each); in one isolate, no carbapenemase was detected (Steinmann et al., unpublished data).

Furthermore, 0.5% (0.1–2.0) of the patients who were tested positive for *Enterobacterales* and/or *A. baumannii* with CR did not report any of the listed characteristics (Table 1 (Tab. 1)). Based on this finding, this cohort should be evaluated in more detail in the future for other potential risk factors. For instance, because aquatic environments and seafood have been shown to be potentially contaminated with fecal indicators, such as carbapenem-resistant bacteria [38], [39], [40], outdoor activities such as rowing, canoeing, diving, snorkeling or consumption of seafood might be risk factors for acquiring colonization or infections by MDRGN. This was reported during the Olympic Games in Rio de Janeiro in 2016 [41], [42], [43].

Considering the high number of reported stays in hospital within the previous 12 months of patients who were tested positive for *Enterobacterales* and/or *A. baumannii* with CR, this factor should be a standard item in the patient’s anamnesis and is suggested to be included future considerations on infection control strategies in hospitals.

Clearly, our study has two major limitations. Since the analysis was based on cases for which notification to the Public Health Agency is legally required, the comparison to a control group is nearly impossible. Theoretically, such a control group should include individuals with the risk factors mentioned above but who were tested negative for *Enterobacterales* and/or *A. baumannii* with CR. These combinations, however, are not systemically recorded for patients at UHF, which in turn restricts the use of statistical tests for significance. Furthermore, the data on anamnestic risk factors obtained by the questionnaire are limited only to previous hospital stays, residential status and country of origin or pre-treatment. These factors are essential to evaluate possible risk factors, but seem to be insufficient in terms of discriminatory power. The official questionnaire would profit from a revision with the intent to find additional, more highly discriminatory questions to identify risk patients carrying MDRGN with CR. For example, additional questions could address the patient’s leisure behavior (e.g., water sports, see above) or profession (e.g., persons with professional contact to waste water). In addition, we found n=99 patients who reported hospitalization abroad within the previous 12 months. Due to language barriers, this group might be misidentified by our questionnaire (which is presented in German). 

In order to facilitate the distinction between “community-acquired” and “hospital-acquired”, KISS recommends determination of the period since patient’s admission to the hospital and set the decisive criterion to three days. Thus, “nosocomial” encompasses cases not detected on admittance, either because

1. they were not screened at that time, or
2. were screened on admittance, but the (low) presence of carbapenem-resistant organisms (CRO) was “masked” by the large amount of other bacteria in the intestinal microbiota and were only unmasked with inception of antibiotic therapy, or
3. CRO were actually generated during antibiotic therapy or, finally
4. CRO were transmitted during a hospital stay.

It is important to realize that only the last case 4 would be sufficient to be influenced by infection control measures. Furthermore, in case of the occurrence of a “resistance plasmid transfer” [44], the label “nosocomial” can also be highly problematic, as the transfer of a resistance plasmid from one species to another (e.g., from an *E. coli* with CR into a carbapenem-susceptible strain of *K. pneumoniae*) might result in the initial detection of a *K. pneumoniae* with CR [44], for instance, which might be misinterpreted as a new nosocomial acquisition. It has been previously shown that resistant strains which are present in the gastrointestinal tract are not detectable via conventional microbiological methods due to their low number. During antimicrobial therapy, these bacteria will be selected, bloom and colonize the intestine [45]. In cases of colonizing organisms with possibly lower screening sensitivity, the current scope of this definition for surveillance according to KISS and Centers for Disease Control and Prevention (CDC) does not completely match the complex coherences in terms of epidemiology, infection control, microbiological and public health. The criteria for using the category “nosocomial” should therefore be reviewed in order to adequately address these complex circumstances. 

## Conclusions

*Enterobacterales* and *A. baumannii* with carbapenem resistance are a major global threat in terms of infection prevention and public health. Regarding the cohort described here, hospital stay within the previous 12 months, including hospitalization abroad as well as in Germany, was the most frequently reported anamnestic factor (75.5% patients reported at least one hospital stay within the previous 12 months). Hospital stay within the previous 12 months therefore is a key anamnestic predictor for carrying *Enterobacterales* and/or *A. baumannii* with CR. Patients should be regularly asked about this anamnestic factor on the day of admission. In order to improve infection control efforts, patients reporting any hospital stay within the previous 12 months should be screened, e.g. by rectal swabs, for *Enterobacterales* and *A. baumannii* upon admission and pre-emptive isolation should be considered. With regard to MDRGN + CR, the criteria characterizing “nosocomial” need to be reviewed.

## Notes

### Competing interests

The authors declare that they have no competing interests.

### Ethical approval

This study was approved by the Ethics Board of the University Hospital Frankfurt, Germany (votum # E151/17). 

## Figures and Tables

**Table 1:**
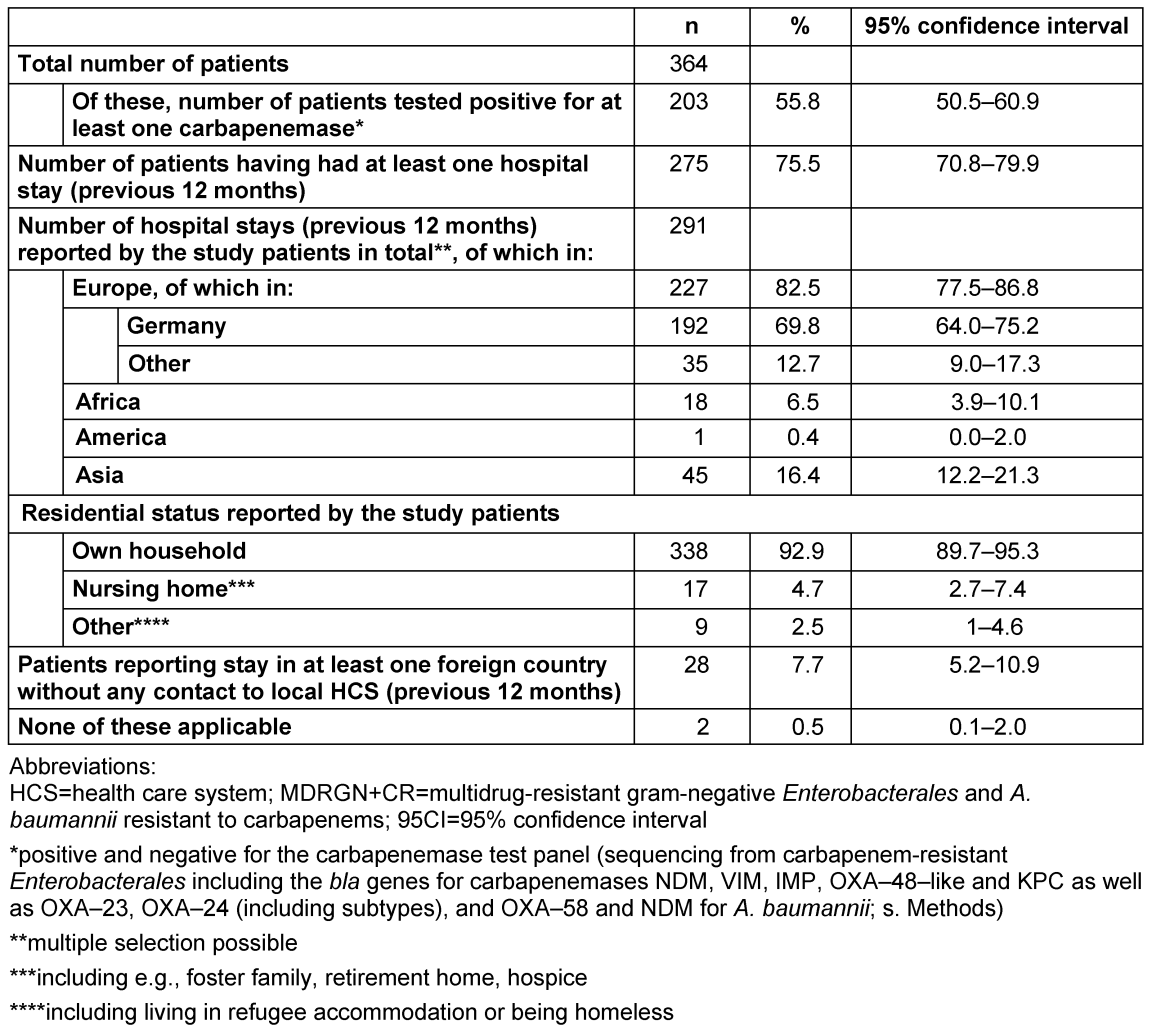
Anamnestic factors reported by patients who tested positive for *Enterobacterales* and/or* A. baumannii* with CR; selection of multiple items was possible
